# A systematic review of non-invasive biomarkers for seizure forecasting in pediatric epilepsy patients

**DOI:** 10.3389/fneur.2026.1780222

**Published:** 2026-04-20

**Authors:** Christine Esparza, Megan Thomas Hebdon, Jun Wang, Chumeng Wang, Rania Agrawal, Grace Do, Avery Bodden, Galilea Dupree, Jacqueline Rajotte, Gabriella Gonzalez Ciofuli, Halena Rios, Yingchao Yuan, Clifford Calley

**Affiliations:** 1 Baylor College of Medicine, Houston, TX, United States; 2 University of Texas School of Nursing, Austin, TX, United States; 3 University of Utah College of Nursing, Salt Lake City, UT, United States; 4 Department of Speech, Language, and Hearing Sciences, University of Texas, Austin, TX, United States; 5 Department of Neurology, University of Texas at Austin Dell Medical School, Austin, TX, United States; 6 Barry University, St. Petersburg, FL, United States; 7 University of Texas, Austin, TX, United States

**Keywords:** algorithms, biomarkers, epilepsy, seizure forecasting, wearable electronic devices

## Abstract

**Introduction:**

Epilepsy is one of the most common neurological disorders globally. While medications, surgical interventions, and dietary changes can be successful in controlling seizures, a subset of individuals experience refractory epilepsy and are at increased risk for sudden unexpected death in epilepsy (SUDEP). Efforts to provide a detection system using devices have been successful at identifying seizures once they start, but there are no devices or systems on the market that predict seizures. The purpose of this systematic review (PROSPERO ID: CRD42024444250) is to determine non-invasive physiologic and environmental biomarkers that can be used to forecast seizures in pediatric epilepsy patients.

**Methods:**

A systematic search of relevant literature was conducted in PubMed, Web of Science, CINAHL Ultimate, and EMBASE in August 2023. Articles were reviewed by two investigators in a two-step process. Data extraction occurred using two independent extractors to identify study characteristics, patient characteristics, and forecasting results. Evidence quality was evaluated by two investigators using the Grading of Recommendations, Assessment, Development, and Evaluation (GRADE) tool.

**Results:**

Eleven observational cohort studies were included and cardiovascular biomarkers using electrocardiogram (ECG) measurement were most commonly used. Pre-ictal anticipation algorithm times ranged from 21.8 s to 32 min, while correlational studies observed cardiovascular biomarker changes 3.59 s to 40 min before seizures. This systematic review provides a comprehensive overview of the current evidence for seizure forecasting. However, the evidence in 9/11 of reviewed studies were rated as either low or very low certainty using the GRADE tool due to methodological flaws, risk of bias, inconsistent results, and indirect or sparse evidence.

**Discussion:**

There are ongoing opportunities to build on our findings, including further testing of cardiovascular biomarkers with other physiologic and environmental factors, larger sample size studies, and a precision medicine approach to tailoring algorithms and biomarker measurements to individual patients.

**Systematic review registration:**

PROSPERO (ID: CRD42024444250).

## 1 Introduction

Epilepsy is a neurological disorder characterized by abnormal electrical discharges in the brain resulting in seizures (1). According to the World Health Organization in 2024, approximately 50 million people globally are affected by epilepsy, making it one of the most common neurological disorders for both adults and children (2, 3). Many patients diagnosed with epilepsy are treated with antiseizure medications; however, about 20–40% of these patients are refractory to medical management, meaning their seizures cannot be controlled with medication alone (4). If these patients are not surgical candidates, if surgery fails to fully control seizures, or if surgery is not desired by the patient and their family, then few options remain to achieve seizure control.

People with epilepsy (PWE) are up to three times more likely than the general population to experience a premature death (2). A 2023 review reported that 22–45% of epilepsy-related deaths in pediatric patients were due to sudden unexpected death in epilepsy (SUDEP), injuries, or drowning (5). Refractory epilepsy is a known risk factor for SUDEP, which accounts for up to 50% of deaths within this group.

Therefore, PWE experience not only seizures but the characteristic uncertainty of epilepsy as well (6). PWE are also more likely than the general population to experience psychological issues such as anxiety and depression (2). These factors compound with treatment side effects, lifestyle restriction, and stigmatization to account for significantly reduced quality of life (7–10). Furthermore, this burden can extend to caregivers of PWE. Studies have shown that caregivers for PWE have negative psychological and physical effects, especially caregivers of children (11). High rates of disease burden for caregivers of PWE are likely attributable to the fear of seizures or a seizure-related incident. The lack of available treatment options and high risk of epilepsy-related deaths for patients with refractory epilepsy may provoke feelings of uncertainty, isolation, frustration, and fear. Due to these factors, this population is likely to significantly benefit from a reliable seizure forecasting strategy.

Seizure forecasting is an area of study that aims to diminish the unpredictability of epilepsy, thereby improving the quality of life for both PWE and their caregivers. It may also help identify unrecognized patterns of seizure onset that can lead to improved medical management. In a 2021 survey of caregivers for PWE, 74% of respondents reported that a device to forecast seizures was extremely important for the epilepsy community. Furthermore, 76% of respondents reported they would use a device that predicts times of high and low seizure risk (12).

Considerable work has been done to predict seizures using scalp and intracranial EEG measurements with varying levels of success (13–19). However, the practical application of EEG seizure prediction remains challenging. For a forecasting device to be useful and effective, it must be as minimally invasive as possible so that it does not hinder a patient from completing activities of daily living. To reach this goal, there is a need to first identify reliable, easily measurable, non-invasive biomarkers with seizure-predictive capabilities. The aim of this systematic review is to generate a comprehensive list of non-invasive biomarkers that can be used for seizure forecasting. Our research question is as follows: In pediatric epilepsy patients, what non-invasive physiologic and environmental biomarkers can be used to forecast seizures before onset?

## 2 Methods

**Figure 1:**
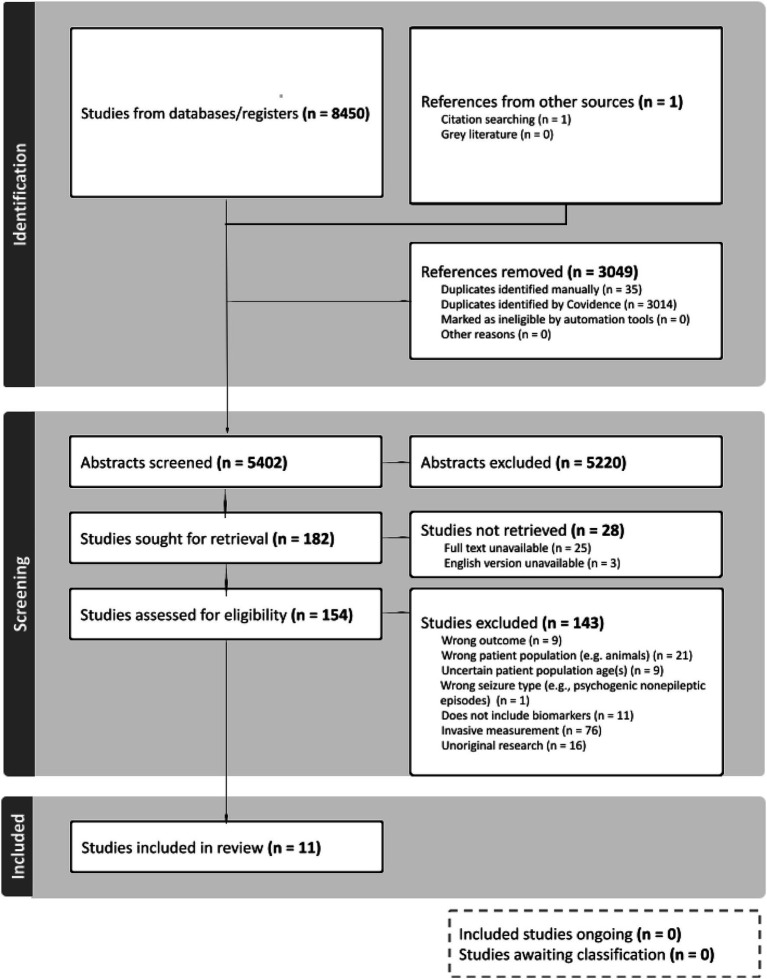
Preferred reporting items for systematic reviews and meta-analyses (PRISMA) flowchart. This chart shows the number of articles that were initially identified from four databases—PubMed, Web of Science, CINAHL Ultimate, and EMBASE (*n* = 8,450)—and citation searching (*n* = 1). Duplicates were removed automatically via Covidence and manually (*n* = 3,049). A total of 5,402 abstracts were screened, then 154 full-text studies were screened, and 11 studies remained for inclusion. Exclusion decisions by number of studies are provided in the flowchart.

**Figure 2:**
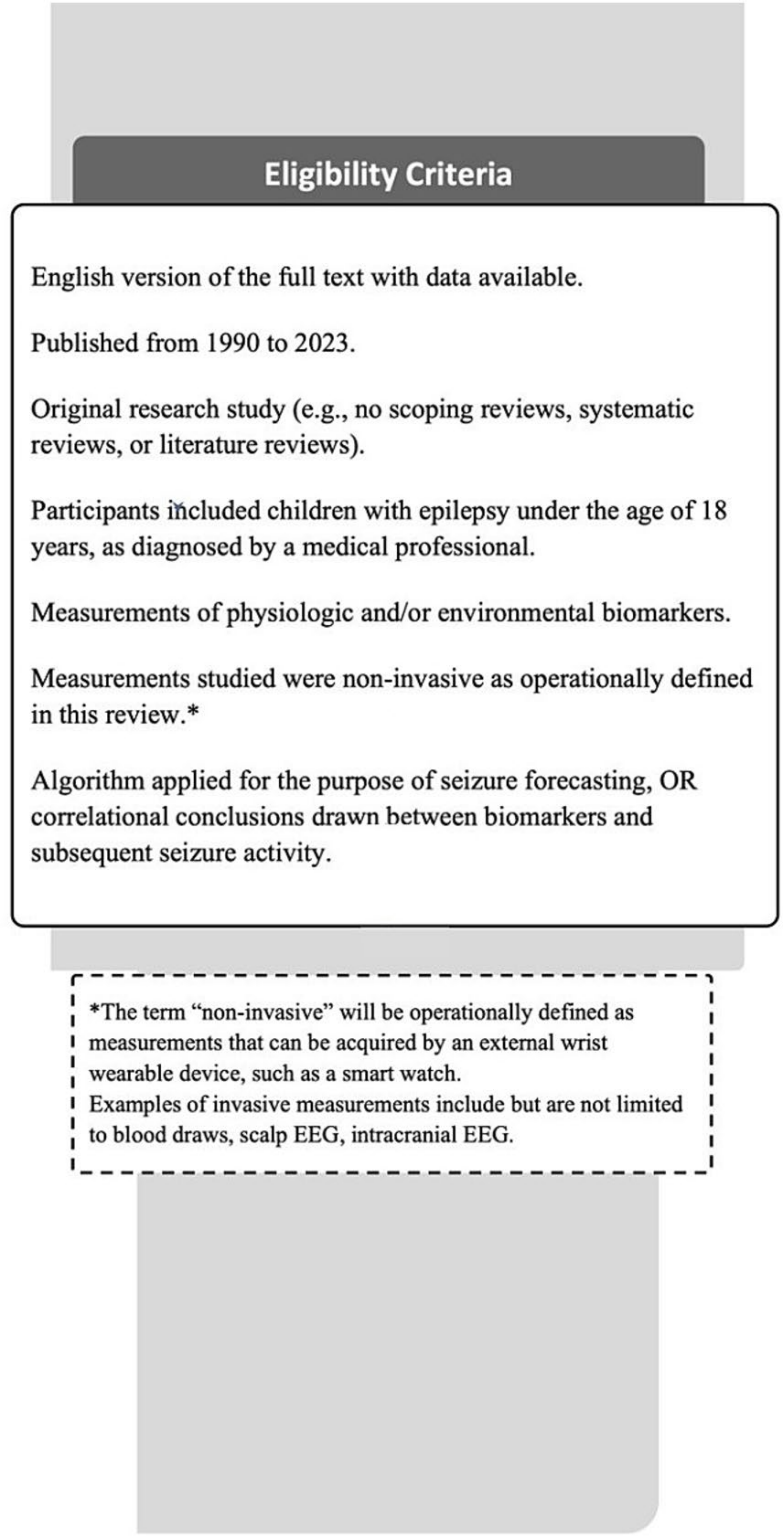
Eligibility criteria. This figure provides the details of this review’s eligibility criteria that each study had to meet for inclusion in the review, along with this review’s operational definition of “non-invasive”.

The protocol for this systematic review was registered on PROSPERO (ID: CRD42024444250), and Preferred Reporting Items for Systematic Review and Meta-Analyses (PRISMA) 2020 item checklist was used to ensure that this review conformed to the most recent guidelines. Each article had to meet the following requirements, summarized in Figure 1, to be selected for inclusion in the review. Studies that were excluded did not meet one or more of these criteria (Figure 2). The search strategy was initially developed on PubMed, then adapted for other electronic databases including Web of Science, CINAHL Ultimate, and EMBASE. The search criteria inputted within PubMed was as follows:

((((Forecasting[MeSH Terms]) AND ((Seizures[MeSH Terms]) AND (Epilepsy[MeSH Terms]))) OR ((Wearable Electronic Devices[MeSH Terms]) AND ((Seizures[MeSH Terms]) AND (Epilepsy[MeSH Terms])))) OR ((Biomarkers[MeSH Terms]) AND ((Seizures[MeSH Terms]) AND (Epilepsy[MeSH Terms])))) OR ((Algorithms[MeSH Terms]) AND ((Seizures[MeSH Terms]) AND (Epilepsy[MeSH Terms]))).

A date filter of 1990–2023 was then used to filter the search. This search strategy was then replicated in each of the other databases used. Date limits of 1990–2023 were informed by a preliminary literature review conducted by the authors which revealed that most literature relevant to the field of seizure forecasting was likely to be found in or after the 1990s. Results from each database were exported to Covidence in August of 2023 for screening, which was split into two phases: title/abstract screening, then full-text screening. After duplicates were removed, 5,402 articles remained for screening by a group of 10 independent reviewers who were trained on the eligibility criteria. Each abstract was screened by two independent reviewers who voted to include or exclude based on the eligibility criteria. Following this phase, 154 articles remained for the full-text screening, in which two independent reviewers assessed each article then voted to include or exclude. If conflicts were identified during both screening phases, a third investigator was invited to independently review and resolve disagreements. Unoriginal research studies (e.g., systematic or scoping reviews) were reference-checked, and through this process, one relevant publication was identified. Following the two screening phases, 10 studies were included from databases and one study was identified through reference checking, leading to a total of 11 studies included in this review. Of these 11 studies, those that were not already reference-checked were screened for additional relevant publications, and none were found.

Data items in this review were manually extracted via synthesis tables by a team of eight independent co-investigators. Each study was reviewed for relevant data items by two or three independent co-investigators. Since this study group had a specific interest in pediatric data, the co-investigators responsible for extracting data were instructed to carefully search for and document study results that were specific to pediatric patients, when present. In the case of a mixed sample including adults and children in which there was no reported pediatric-specific data, the co-investigators were instructed to report results since they were influenced by pediatric data. Data items were split into categories of study characteristics, patient characteristics, and forecasting results. Items under patient characteristics included sample size, age range, mean age, gender, seizure type(s), and seizure frequency. Within the category of study characteristics, the data items recorded were length of study time, setting, biomarkers studied, method of biomarker measurement, method of seizure activity measurement, and predictive models used, if any. Each of these data items contributed to the evidence grade.

During data extraction, evidence was graded using the Grading of Recommendations, Assessment, Development, and Evaluation (GRADE) system and a two-step process to promote reliability, with an initial rating identified by one of two independent investigators and then checked and revised by a senior author (20). A meta-analysis was not conducted due to methodological and statistical heterogeneity. The GRADE system uses four categories on a continuum: high, moderate, low, and very low. Rather than rating based on specific study types (e.g., automatic high rating for randomized controlled trials), the reviewers walked through an algorithm that outlines when to upgrade or downgrade the evidence rating based on study characteristics (21). A high rating indicates that the study was well-conducted and had consistent results with minimal risk of bias. Moderate certainty indicates that there may be inconsistencies and possible bias. A study rated with low certainty would have methodological flaws, high risk of bias, inconsistent results, and indirect evidence. Very low certainty indicates major methodological limitations, contradictory results, high risk of bias, and sparse evidence (20, 21) (see Tables 1, 2).

**Table 1:** Correlational study details.

Study name	Study type	Average participant age	Sample size and length of study	Seizure type(s)	Biomarkers studied	Findings	Evidence grade
Using methods of time series data mining to recognize the influences of environmental factors on epileptic seizures (2018) (22)	Retrospective cohort study; correlational	Not specified	Not specified. 84 months total	Not specified	Accumulated precipitation, temperature, max and min temperature, relative humidity, max and min relative humidity, mean wind speed and direction, atmospheric pressure, max and min atmospheric pressure, and sunshine duration.	Ambient temperature negatively correlated with seizure occurrence. Max atmospheric pressure positively correlated with seizure occurrence	Very low certainty
Seizure-related differences in biosignal 24-h modulation patterns (2022) (23)	Prospective cohort study; correlational	Median age (IQR) at enrollment: No-seizure group 9.4 (8.4), Seizure group 13.2 (7.2)	117 pediatric*. 24 h per patient.	Focal impaired awareness seizures and generalized tonic–clonic seizures	EDA, TEMP, HR	Decreased HR in pre-ictal period for 14 (29%) pediatric patients compared to ictal period	Moderate certainty
Novel seizure biomarkers in continuous electrocardiograms from pediatric epilepsy patients (2021) (24)	Retrospective cohort study; correlational	Median = 10.2 y, IQR = 6.2 y	22 pediatric*. 17.7 to 227.4 h per patient. Median 93.3 h.	Focal seizures	HR, RRI, FD, ApEn	68.2% of patients showed changes in at least one feature, including 40.9% with changes in HR/RRI between no-seizure and seizure segments	Low certainty
Peri-ictal ECG changes in childhood epilepsy: implications for detection systems (2013) (25)	Retrospective cohort study; correlational	9.2 y (no SD provided)	35 pediatric*. 24 h per patient	Focal seizures (TLE, frontal lobe) and generalized seizures (tonic, tonic–clonic, and myoclonic)	HR	Pre-ictal HR changes in 70% of focal seizures. Average time from HR change to seizure = 3.59 s (0.2 to 29 s). Sensitivity 43%, specificity 95%	Low certainty
Heart rate variability analysis for the identification of the preictal interval in patients with drug-resistant epilepsy (2021) (27)	Retrospective cohort study; correlational	41 ± 16 y	41 total**. Length of study unspecified	TLE	HRV	HRV features characterizing pre-ictal period seen in 41% of seizures and 90% of patients. Most changes seen within 40 min of seizure onset	Low certainty
Monitoring heart period variability changes during seizures II. Diversity and trends (1996) (29)	Retrospective cohort study; correlational	24.1 y; pediatric-specific data not given	12 total**. Length of study unspecified	Complex partial seizures, tonic–clonic seizures	HR, HRV	Pre-ictal tachycardia in 16% of seizures	Very low certainty
Autonomic nervous system changes detected with peripheral sensors in the setting of epileptic seizures (2020) (31)	Prospective cohort study; correlational	9.9 ± 5.8 y	66 total (62 pediatric). 44 mo total	Focal and generalized onset seizures	EDA, TEMP, HR	Pre-ictal EDA signal entropy increased for 17 patients (*p* < 0.05)	Very low certainty

ACC, accelerometer data in three dimensions; ApEn, approximate entropy; BVP, blood volume pulse; EDA, electrodermal activity; FD, fractal dimension; h, hours; HR, heart rate; HRV, heart rate variability; min, minutes; mo, months; RRI, R-to-R interval; s, seconds; TEMP, peripheral body temperature; TLE, temporal lobe epilepsy; y, years. *Pediatric patients only in the study. **Pediatric-specific data not specified. ACC, accelerometer data in three dimensions; ApEn, approximate entropy; BVP, blood volume pulse; EDA, electrodermal activity; FD, fractal dimension; h, hours; HR, heart rate; HRV, heart rate variability; min, minutes; mo, months; RRI, R-to-R interval; s, seconds; TEMP, peripheral body temperature; TLE, temporal lobe epilepsy; y, years. *Pediatric patients only in the study. **Pediatric-specific data not specified.

**Table 2:** Algorithm study details.

Study name	Study type	Average participant age	Sample size and length of study	Seizure type(s)	Biomarkers studied	Findings	Evidence grade
Focal epileptic seizures anticipation based on the patterns of heart rate variability parameters (2019) (26)	Prospective cohort study; algorithm testing	8.2 ± 4.3 y	9 pediatric*. 12–24 h per patient	Focal seizures	HRV	Anticipation accuracy 77.1%. Anticipation time of 21.8 s	Very low certainty
Patient-specific seizure prediction based on heart rate variability and recurrence quantification analysis (2018) (28)	Mixed retrospective and prospective cohort study; algorithm testing	17.6 ± 9.9 y	15 total (9 pediatric). Length of study unspecified	Focal impaired awareness, generalized onset motor, focal to bilateral tonic clonic	HR, HRV	Experiment 1: Accuracy 88.86% Sensitivity 89.06% Specificity 89.34% False positive rate 0.41/h Pre-ictal period defined as 15 min before a seizure Experiment 2: Accuracy 74.62% Sensitivity 70.16% Specificity 76.98% False positive rate 3.4/h Anticipation time 13.7 min	Low certainty
Wearable epileptic seizure prediction system with machine-learning-based anomaly detection of heart rate variability (2020) (30)	Prospective cohort study; algorithm testing	28 y (No SD provided)	7 total (2 pediatric) Length of study unspecified	Focal seizures (impaired awareness and aware), and focal to bilateral tonic clonic	HRV	Sensitivity 85.7% False positive rate 0.62/h, not significantly different from healthy controls Predicted seizures within 15 min	Low certainty
Machine learning from wristband sensor data for wearable, noninvasive seizure forecasting (2020) (32)	Prospective cohort study; algorithm testing	9.8 ± 5.9 y	69 total (66 pediatric) 2311.4 h total	Focal onset, primary and/or secondary generalized onset, and/or unclassified	EDA, ACC, BVP, TEMP	Accuracy better than chance in 43.5% of patients. Sensitivity 51.2% Mean prediction horizon 1844 ± 80 s	Moderate certainty

ACC, accelerometer data in three dimensions; BVP, blood volume pulse; EDA, electrodermal activity; h, hours; HR, heart rate; HRV, heart rate variability; min, minutes; s, seconds; TEMP, peripheral body temperature; TLE, temporal lobe epilepsy; y, years. *Pediatric patients only in the study. **Pediatric-specific data not specified.

## 3 Results

Table 3 details the sample size and patient demographics for those analyzed in each study. All identified studies included pediatric patients in the sample. Some studies were exclusive to pediatric patients (4/11), while others represented adults and children (7/11). A range of 2 to 117 pediatric epilepsy patients were studied. Studies including both adults and children had smaller average sample sizes (n = 35), while those exclusive to pediatric patients had larger sample sizes on average (n = 46). Eight studies reported a mean age of their sample, which yielded an average patient age of 18.5 years. Many of these studies included adult data as well. One study did not provide information on the average age of the sample (22), while the remaining two provided median measures (23, 24). One of which was exclusive to 117 pediatric patients (23), and the last reported a median age of 10.2 years (24). We extracted all pediatric-specific data reported from each of the studies; however, some of these studies did not report pediatric-specific data (Table 3). Data and takeaways from these studies were still analyzed in this review because pediatric patients were represented. Of note, one retrospective cohort study reported the number of seizures (80,554 total) instead of sample size over a 6-year period (22). The study team then stratified the total seizures by age group for analysis, including the 0-10-year-old age group, which accounted for 11% of total seizures.

**Table 3:** Algorithm study results summary.

Study name	Biomarkers studied	Accuracy (sensitivity, specificity)	Prediction time	False positive rate
Focal epileptic seizures anticipation based on the patterns of heart rate variability parameters (2019) (26)	HRV	Accuracy 77.1%.	Anticipation time of 21.8 s	Not reported
Patient-specific seizure prediction based on heart rate variability and recurrence quantification analysis (2018) (28)	HR, HRV	Experiment 1: Accuracy 88.86% Sensitivity 89.06% Specificity 89.34% Experiment 2: Accuracy 74.62% Sensitivity 70.16% Specificity 76.98%	Experiment 2: Anticipation time 13.7 min	Experiment 1: 0.41/h Experiment 2: 3.4/h
Wearable epileptic seizure prediction system with machine-learning-based anomaly detection of heart rate variability (2020) (30)	HRV	Sensitivity 85.7%	Within 15 min	0.62/h
Machine learning from wristband sensor data for wearable, noninvasive seizure forecasting (2020) (32)	EDA, ACC, BVP, TEMP	Accuracy better than chance in 43.5% of patients. Sensitivity 51.2%	Mean prediction horizon 1844 ± 80 s	Not reported

ACC, accelerometer data in three dimensions; BVP, blood volume pulse; EDA, electrodermal activity; h, hours; HR, heart rate; HRV, heart rate variability; min, minutes; s, seconds; TEMP, peripheral body temperature.

Table 3 lists notable characteristics for each study reviewed, including the study type, length, biomarker choice, and anticipation time/success rate for those that tested a forecasting algorithm. All 11 studies included in this review were cohort studies. Five studies were prospective, five studies were retrospective, and one study had a mixed prospective and retrospective design. One pediatric-specific study that included 22 patients analyzed each patient for 17.7 to 227.4 h (median 93.3 h) (24). The longest study was a retrospective cohort study, which analyzed environmental biomarkers and collected data from January 2007 to December 2013.22 Three studies’ analyses ranged from 12 to 24 h per patient (23, 25, 26), while specific durations were not reported in others (27–30).

Ten of 11 studies reviewed here analyzed physiological cardiovascular biomarkers for use in seizure forecasting. These were most frequently measured using either continuous electrocardiogram (ECG) monitoring or the Empatica E4 biosensor wristband. The biomarkers extracted from ECG measures included heart rate (HR), heart rate variability (HRV), R-to-R interval (RRI), fractal dimension (FD), and approximate entropy (ApEn). Additional physiological biomarkers, such as peripheral body temperature (TEMP), blood volume pulse (BVP), accelerometer data (ACC), and electrodermal activity (EDA), were also measured using the Empatica E4 wristband. Environmental biomarkers were represented by one study that assessed the accumulated precipitation, temperature, relative humidity, mean wind speed and direction, station pressure, and sunshine duration and their relation to seizure activity (22). Each study reviewed fell into one of two categories: those that aimed to observe biomarker changes preceding seizure activity (22–25, 27, 29, 31) and those that aimed to test a newly developed forecasting algorithm using selected biomarkers (26, 28, 30, 32).

Multiple of the studies that tested a forecasting algorithm reported high levels of forecasting accuracy using similar ECG features (26, 28). The former studied 42 focal seizures in nine pediatric patients. The HRV features that increased preceding seizure activity in this study were the mean HR, standard deviation of RRI (SDRRI), power in low-frequency band (LF), LF/HF, and ECG time series upper envelope. The HRV features that decreased before seizure onset were the mean RRI and power in high-frequency band (HF). Using these HRV measurements in addition to others, each patient was given a unique threshold based on their baseline measurements. The resulting anticipation algorithm achieved a 77.1% accuracy with an average anticipation time of 21.8 s preceding seizure activity (26). A similar study that aimed to create a patient-specific seizure algorithm analyzed a sample size of 15 patients (nine pediatric). Of all seizures, pediatric seizures accounted for 24/38 (63%). Similarly, this study also found a decrease in mean RRI (increase in mean HR) and an increase in LF/HF in the pre-ictal period. One finding unique to this study was a decrease in the number of successive pairs that differ more than 50 ms (RRI50) pre-ictally for patients with temporal lobe epilepsy (TLE). This study consisted of two experiments, the first of which aimed to identify a pre-ictal period defined as 15 min preceding seizure activity and achieved an accuracy of 88.86%, a sensitivity of 89.06%, a specificity of 89.34%, and a false positive rate of 0.41/h. A patient-specific algorithm selected features for each of the 15 patients, with an average of 6.7 features selected per patient. In the second experiment, six patients who had three or more seizures throughout the duration of the study were each used to train and test the algorithm, which achieved an average prediction time of 13.7 min with a higher false positive rate of 3.4/h and an average accuracy, sensitivity, and specificity of 74.62, 70.16, and 76.98%, respectively (28). Applied statistics in machine learning were used in one study to demonstrate how changes in RRI extracted from ECG measurements in the pre-ictal period could be used to achieve a seizure prediction sensitivity of 85.7% in 12/14 seizures, including five pediatric seizures, with a false positive rate of 0.62/h (30). Notably, 2/7 of the total patient sample size were pediatric patients, and it was not stated whether RRI increased or decreased in the pre-ictal interval. The fourth algorithm study used the Empatica wrist-watch to study a sample size of 69 patients, of which 66 were pediatric. Using the wrist wearable to measure EDA, TEMP, BVP, and ACC, this team was able to achieve a forecasting accuracy better than chance in 43.5% of all patients, with a mean prediction time of 1844 +/− 80 s (29–32 min). Sensitivity for all patients was 51.2%, and performance metrics improved as more patients were included in the training dataset. The prediction algorithm performed best with all biomarkers used in combination (32). A similar profile of biomarkers were studied in 117 pediatric patients (49 of which had seizures) for correlations of EDA, TEMP, and HR changes in the pre-ictal and ictal periods (23). The team reported a decreased trend in EDA amplitude and lower HR in the pre-ictal period in 14 patients (29%) compared to the ictal period, although the former did not reach significance (*p* = 0.09). Alternatively, a prospective observational cohort study of 66 patients, of which 62 were pediatric, assessed the same biomarkers (EDA, TEMP, HR) and calculated the mean, variance, and entropy of each biomarker. Entropy of EDA was found to increase between the interictal and pre-ictal period for 17/66 (26%) patients (*p* = 0.025). All other biomarker changes were statistically insignificant between the interictal and pre-ictal periods (31).

HRV measures were a common physiologic cardiovascular biomarker among multiple correlational studies, including one that analyzed pre-ictal HRV for a total of 238 seizures from 41 patients, including adults and children with TLE (27). HRV changes occurred in 41% of seizures and 90% of patients in the sample and were mostly observed within 40 min before seizure onset. These results were achieved by clustering multiple HRV parameters such as time domain features related to the RRI (RRImax, RRImin, and RRImean), and other measures, some of which were mentioned in other studies: LF/HF, pRRI50, RRI50, and nonlinear recurrence quantification analysis (RQA ENT). One study did not define a “pre-ictal window” but instead analyzed biomarker changes in segments with and without seizures (24). In this pediatric-specific study (n = 22), ECG was measured to obtain ApEn, FD, RRI, and HR. Sixty-eight percent of patients showed changes in at least one of the features between seizure and nonseizure segments. Eight patients had increased HR and one had decreased HR in seizure segments compared to nonseizure segments (*p* < 0.05). Three patients had increased ApEn, while one had decreased ApEn in seizure segments compared to nonseizure segments (*p* < 0.04). Lastly, four patients had increased FD, and one patient had decreased FD in seizure segments compared to nonseizure segments. However, this study team described these changes as “peri-ictal,” and did not distinguish between pre-ictal and post-ictal states.

HR changes preceding seizure activity were presented with inconsistent findings across studies. One study analyzing 44 seizures from 12 patients observed pre-ictal HR and HRV changes in 16% of seizures, which were described as pre-ictal tachycardia (29). Another retrospective cohort study that was exclusive to 35 pediatric patients observed HR changes in 70% of focal seizures in the pre-ictal period, in which 62% exhibited pre-ictal tachycardia, while the remaining 8% exhibited pre-ictal bradycardia. The average time before seizure onset that these changes were observed was 3.59 s, with a range of 0.2–29 s. Mean HR changes in this study achieved a sensitivity of 43% and a specificity of 95% (25).

Only one study based out of Taiwan observed correlations between meteorological factors and seizure activity (22). The age groups assessed in the study were 0–10, 21–30, 31–40, and 41–50 years. In the pediatric age group, 0–10 years, seizure occurrence significantly increased in January and February. Increased seizure incidence in January was similarly seen in adults aged 21–50 years as well. Among all meteorological factors, maximum and minimum temperature, also known as ambient temperature, was negatively correlated with epileptic seizures in 10 out of 12 geographic regions in Taiwan (CC –0.407 to −0.224, *p* < 0.05). Alternatively, an increase in maximum atmospheric pressure was positively correlated with increased seizure counts in seven out of 12 regions in Taiwan (CC 0.218 to 0.328, p < 0.05). There was no pediatric-specific data given for these biomarkers.

## 4 Discussion

Identifying biological and environmental predictors of seizures non-invasively has tremendous potential to reduce morbidity and mortality in PWE. The two main study purposes identified in this review are correlational studies and algorithm testing studies, and each provides valuable information about potential clinical utility in seizure forecasting. Cardiovascular features measured by ECG, such as HRV and HR/RRI, were the most widely utilized biomarkers studied in articles comprising this review, followed by electrodermal activity, body temperature, ambient temperature, and atmospheric pressure.

Multiple studies achieved moderate to high accuracy in their forecasting algorithm using HRV features. Two studies showed a decrease (26, 28). Despite this similarity, their anticipation times differed significantly, with one reporting an average of 21.8 s, while the other reported 13.7 min (26, 28). The former, which anticipated seizure occurrence much closer to the event, identified that the HRV features that increased in the pre-ictal period were HR, SDRRI, HRstd, total spectral power, LF, LF/HF, LFnorm, and ECG time series upper envelope, while the HRV features that decreased before seizure onset were RRI, HFnorm, and pRRI50. The latter indicated an increase in LF/HF and a decrease in RRI, HF, and RRI50 in the pre-ictal period. However, the decrease in pre-ictal HF and RRI50 were unique to patients with TLE. This suggests that some seizure forecasting biomarkers may be more effective for certain seizure types, supporting the utility of an individualized seizure forecasting algorithm. The two studies reported similar forecasting accuracies (74.62 and 77.1%) with a wide range in anticipation times. A longer anticipation time gives the patient more time to prepare for seizure onset. Therefore, 13.7 min could be preferred for some patients compared to 21.8 s. However, a high false positive rate of 3.4/h was unique to the experiment that anticipated seizures 13.7 min before they occurred. Notably, this study team also conducted a different experiment that used biomarkers to delineate between interictal and pre-ictal periods with a false positive rate of 0.41/h (accuracy 88.86%, sensitivity 89.06%, specificity 89.34%). Correctly identified pre-ictal periods were within 15 min of seizure onset. These experiments support the use of patient-specific forecasting algorithms. However, the high false positive rates in both of these experiments heavily weaken the impact of the model’s accuracy and early anticipation. Similarly, an additional algorithm testing study reported a false positive rate of 0.62/h, which correlates to approximately one false positive alarm every 2 h (30). Devices with high false positive rates such as these lack clinical utility because they can cause unnecessary disruption in the lives of patients and caregivers, leading them to stop using the device (33).

The third study that tested a forecasting algorithm assessed different biomarkers (EDA, TEMP, BVP, ACC) and achieved a forecasting accuracy better than chance for less than half of the patients (*n* = 69). While this algorithm did not perform as well as the former two, the sample size of this study was much larger, and the mean prediction time was longer as well, approximately 30 min (32). Additionally, one prospective cohort study identified a decrease in HR in the pre-ictal period compared to the seizure period for 14 patients (29%), while pre-ictal to ictal EDA changes did not reach significance (23). A different prospective cohort study found significant changes in EDA in the pre-ictal period, as the entropy calculation of EDA increased from the interictal period to the pre-ictal period in 17 patients (*p* = 0.025). However, HR and TEMP did not show a significant difference between the interictal and pre-ictal periods (31). EDA is a physiological measurement of skin conductance and is mediated primarily by the sympathetic branch of the autonomic nervous system and associated with the physiological “fight-or-flight” response (34). Simultaneous bradycardia and increased EDA amplitude suggests concurrent activation of both the sympathetic and parasympathetic nervous system in the pre-ictal period (23). Although these studies did not all agree with the utility of these biomarkers, they all point to the similar key physiological mechanism, autonomic nervous system imbalance, that may underlie the pre-ictal period for some patients. These inconsistent findings also support the potential utility of an individualized algorithm for forecasting rather than a one-size-fits-all strategy.

Other observational studies aimed to find correlations between cardiovascular features and subsequent seizure occurrence (23–25, 27, 29, 31). These studies mainly focused on features derived from HRV, including HR, RRI, LF/HF, pRRI50, RRI50, approximate entropy, fractal dimension, and RQA ENT. One study assessed changes in HR alone, using RRI as a surrogate data marker (25). HR changes were detected in the pre-ictal period of 70% of focal seizures for 35 pediatric patients. However, these changes were seen only in focal seizures and not in generalized seizures in this sample. Pre-ictal HR changes included both tachycardia and bradycardia, although pre-ictal tachycardia represented the overwhelming majority of patients. This study did not specify how they defined tachycardia or bradycardia, which would have been useful given the differing ranges of normal HR across the included age ranges. While HR changes achieved a high specificity of 95%, the sensitivity was low at 43%. Since a seizure forecasting mechanism aims to alarm the user that there is a chance of an impending seizure, a higher sensitivity is preferred, making HR changes alone a less optimal biomarker in this study. This supports the conclusion that one biomarker may not be effective for seizure forecasting when used alone, and that combinations of biomarkers may be preferable. A different retrospective cohort study defined tachycardia as a HR increase of greater than 15 beats per min, which was identified in 16% of 44 seizures among 12 patients. It is important, however, to note that the number or age of pediatric patients in this sample was unspecified; only the age range of 4.5–55 years with a mean of 24.1 years was given.29 Due to the wide age range, mean of greater than 18 years, and small effect of 16% of seizures exhibiting changes in the pre-ictal period, the findings of this study are less impactful than those more directly applicable to the population being studied for this review. Another pediatric-specific study observed ECG changes in ApEn, FD, RRI, and HR, in which 68.2% of patients showed changes in at least one of the features between the segments containing seizures and those without. However, a specific “pre-ictal” phase was not evaluated in this study, so conclusions that can be drawn for this review are highly limited due to the uncertainty of timing reported. Furthermore, although the team stated “electrocardiographic changes occur frequently in patients during seizure evolution, in some cases hours before ictal onset,” they did not expand on this finding. There is a high level of inconsistency among the literature regarding the utility of pre-ictal HR changes as a biomarker for seizure forecasting. Patients in some studies have presented with pre-ictal HR increases, while others have shown HR decreases, and many with no change. This conclusion suggests that HR changes may be a useful predictor for some epilepsy patients, while others may benefit from the use of different biomarkers.

Only one study sought to observe meteorological characteristics and their correlation with the increased incidence of epileptic seizures (22). Due to age group stratification and result reporting in this study, pediatric-specific information was analyzed only for the 0–10 age group. No results were reported for the 11–20 age group. Maximum atmospheric pressure was positively correlated with an increased seizure count in seven out of 12 regions in Taiwan, while a negative correlation between ambient temperature and seizure occurrence was found in 10 out of 12 regions, mainly seen in January and February. However, these were weak correlations (CC 0.218 to 0.328 and −0.407 to −0.224, respectively). Furthermore, the winter months are associated with increased rates of viral illnesses, and studies have shown associations between an increased risk of epileptic seizures and time periods of illness, fever, or increased stress (35–38). Notably, this study did not account for concomitant illness in the participants, leading to the possibility of unaccounted confounding factors. The chosen stratification method and how the results were reported by the study team greatly limits use of this evidence in this review. However, this was the only study identified to analyze meteorological characteristics for seizure forecasting, and it had a significantly larger sample size than all other studies identified. Regarding the confirmation of seizure occurrence, surrogate data (i.e., emergency medical records coded as epileptic seizures) were used in place of direct data (i.e., EEG-confirmed seizure activity). Additionally, sample size surrogate data was seizure count rather than number of patients analyzed in this study. Thus, patients with many recurrent seizures could possibly influence any correlations made in a positive or negative manner. Surrogate data for seizure confirmation and sample size are examples of indirectness, which diminishes the reliability of the correlations drawn from this study and weakens the findings.

There is controversy among the literature regarding whether seizure forecasting can be successfully achieved with a one-size-fits-all algorithm or whether individualized algorithm is necessary. Most studies reviewed here present evidence that supports the need for individualized seizure algorithms to achieve accurate seizure forecasting. Evidence for this conclusion is well-demonstrated by the presence of changes in baseline HR in the pre-ictal period. Some patients showed no change in HR during the pre-ictal period, while others experienced a pre-ictal HR increase, and a small subset experienced a pre-ictal HR decrease. Similar inconsistencies were seen with other HRV and meteorological measures. While there were certainly trends in HR and HRV biomarkers, the strongest associations were present in sample sizes with the same type of seizures such as TLE or focal seizures (26–28).

The studies reviewed here regarding pediatric epileptic seizure forecasting mostly rely on pre-ictal fluctuations in cardiovascular features derived from ECG measures. There is variation within this literature about the utility of HR changes in seizure forecasting. Detection of pre-ictal HR changes in some patients suggests that it may be a useful biomarker for those individuals, while others may not benefit from it. EDA was assessed in two studies, but a small percentage of patients showed detectable changes in the pre-ictal period in one study. HRV features were used in multiple studies to achieve a high prediction accuracy or sensitivity (26, 28, 30). However, the false positive rate was also high, and some evidence suggests that this may be most helpful in patients with TLE. One study assessing environmental biomarkers met inclusion criteria, which found negative and positive correlations between ambient temperature and maximum station pressure with seizure count, respectively. However, this study design was susceptible to possible confounding variables, thus weakening the conclusions of this study for the purpose of this review.

Finally, the process of grading the evidence was completed via a two-step process. The initial level of certainty was determined by the study design, in which randomized controlled trials or studies evaluated with ROBINS-I are given an initial grade of high certainty while observational studies are initially given a low level of certainty. All studies reviewed here were observational studies, and thus received an initially low level of certainty. The second step in the process included upgrading or downgrading the level of certainty based on key features such as confounding factors, sample size, the presence of a large effect, and measurement bias. Two studies that were upgraded to moderate certainty were due to larger sample sizes, significance of results, and adjustments for confounding factors. Four studies were downgraded to very low certainty due to small sample sizes, possible confounding factors, and data analysis that was either incompletely reported or inconsistent. As previously mentioned, the one study which reported on environmental biomarkers measured seizure counts through emergency medical records rather than electrographically-confirmed methods, leading to measurement bias, which was a major reason for the downgrade of this study to very low certainty (22). The five remaining studies, including two algorithm studies that achieved high sensitivities, were neither upgraded nor downgraded from low certainty due to small sample sizes and no large effect. Limitations of the studies were primarily due to low sample sizes and short duration of monitoring. The number of pediatric patients reported by the analyzed studies ranged from 2 to 117. Even the study with 117 pediatric patients stated that it was too small of a sample for a machine learning model to accurately identify seizure forecasting trends (23). Small sample sizes indicate a need for future studies with larger, pediatric-specific cohorts over longer periods to properly identify reliable seizure forecasting variables. Algorithms that are trained on larger datasets may also improve forecasting accuracy and minimize false positives. Central to the clinical utility of seizure forecasting devices are the false positive rates. Only two studies reported false positive rates for their forecasting algorithms, both of which were concerningly high (28, 30). The absence of reported false positive rates from other studies greatly weakens the ability for clinical translation of these algorithms. Additionally, a majority of the studies reviewed here were conducted in an epilepsy monitoring unit and are thus not representative of patients’ daily lives. Therefore, reproducibility of these seizure prediction biomarkers outside of a hospital setting must be studied in the future. Other limitations potentially hindering data analysis include the lack of representations of various seizure types, wide pediatric age ranges, difficulty delineating pediatric-specific data from adult data, and pharmacological variations between patients (28, 31). Lastly, these conclusions should be taken with caution, due to the evidence grading of low or very low certainty for nine out of 11 of the studies reviewed here.

Developing an accurate wearable seizure forecasting device would significantly reduce morbidity and mortality and give refractory epilepsy patients and their caregivers more control over their condition. Such a device could both improve quality of life for patients and their caregivers and enhance long-term health outcomes by reducing pediatric deaths associated with epilepsy, including SUDEP. Identifying reliable biomarkers with predictive capabilities through evidence-based investigations is a crucial first step. This review summarizes the known literature on pediatric biomarkers for seizure forecasting and highlights the absence of well-designed studies which are needed to validate specific biomarkers and predictive algorithms. More studies are needed to assess not only each biomarker’s predictive power in isolation but also combinations of biomarkers to increase reliability and accuracy. Additionally, the forecasting capability of each biomarker and/or combinations of biomarkers will likely need to be optimized to the individual patient to achieve reasonable practicability. While these preliminary studies do not present exceptionally strong evidence, they still provide valuable groundwork to guide future research that overcomes the discussed limitations by incorporating larger pediatric cohorts that are followed outside the hospital over longer periods, thereby establishing more robust, evidence-based algorithms for seizure forecasting.

## Data Availability

The original contributions presented in the study are included in the article/supplementary material, further inquiries can be directed to the corresponding author.
